# Dietary Polyphenols, Plant Metabolites, and Allergic Disorders: A Comprehensive Review

**DOI:** 10.3390/ph17060670

**Published:** 2024-05-22

**Authors:** Mohd Farhan, Asim Rizvi, Mohammad Aatif, Ghazala Muteeb, Kimy Khan, Farhan Asif Siddiqui

**Affiliations:** 1 Department of Chemistry, College of Science, King Faisal University, Al Ahsa 31982, Saudi Arabia; 2 Department of Basic Sciences, Preparatory Year, King Faisal University, Al Ahsa 31982, Saudi Arabia; 3 Department of Biochemistry, Faculty of Life Sciences, Aligarh Muslim University, Aligarh 202002, India; rizvirizviasim@gmail.com; 4 Department of Public Health, College of Applied Medical Sciences, King Faisal University, Al Ahsa 31982, Saudi Arabia; maahmad@kfu.edu.sa; 5 Department of Nursing, College of Applied Medical Sciences, King Faisal University, Al Ahsa 31982, Saudi Arabia; graza@kfu.edu.sa; 6 Department of Dermatology, Almoosa Specialist Hospital, Dhahran Road, Al Mubarraz 36342, Al Ahsa, Saudi Arabia; kimykhan@gmail.com; 7 Department of Laboratory and Blood Bank, King Fahad Hospital, Prince Salman Street, Hofuf 36441, Saudi Arabia; farhans@moh.gov.sa

**Keywords:** polyphenols, allergy, asthma, allergic rhinitis, atopic dermatitis, food allergy

## Abstract

Given the ongoing rise in the occurrence of allergic disorders, alterations in dietary patterns have been proposed as a possible factor contributing to the emergence and progression of these conditions. Currently, there is a significant focus on the development of dietary therapies that utilize natural compounds possessing anti-allergy properties. Dietary polyphenols and plant metabolites have been intensively researched due to their well-documented anti-inflammatory, antioxidant, and immunomodulatory characteristics, making them one of the most prominent natural bioactive chemicals. This study seeks to discuss the in-depth mechanisms by which these molecules may exert anti-allergic effects, namely through their capacity to diminish the allergenicity of proteins, modulate immune responses, and modify the composition of the gut microbiota. However, further investigation is required to fully understand these effects. This paper examines the existing evidence from experimental and clinical studies that supports the idea that different polyphenols, such as catechins, resveratrol, curcumin, quercetin, and others, can reduce allergic inflammation, relieve symptoms of food allergy, asthma, atopic dermatitis, and allergic rhinitis, and prevent the progression of the allergic immune response. In summary, dietary polyphenols and plant metabolites possess significant anti-allergic properties and can be utilized for developing both preventative and therapeutic strategies for targeting allergic conditions. The paper also discusses the constraints in investigating and broad usage of polyphenols, as well as potential avenues for future research.

## 1. Introduction

The incidence of allergic illnesses has risen significantly in the last two to three decades, and it is currently impacting not only developed nations but also underdeveloped/developing economies [1,2,3,4,5,6,7,8]. Given the unlikelihood of genetic predispositions being the only factor responsible, it is more appropriate to explain this significant rising trend by alterations in environmental factors. The changes in dietary patterns and practices in recent decades appear to be a credible environmental explanation, as shown by several studies [9,10,11,12,13]. In addition, based on the diet hypothesis, nutrients and food components may have a significant impact on the immune system and pathways related to allergic inflammation [12]. This influence can occur directly or indirectly through the modulation of gut microbiota [14,15]. Consequently, these factors can either contribute to or protect against allergic diseases [14,15,16,17]. Given that diet is a highly changeable and easily available intervention, it is crucial to identify specific nutrients, food components, or dietary patterns that might be used as preventive or therapeutic measures for allergic diseases.

A study group has recently introduced an immune-supportive diet as a dietary intervention to be included in the overall management (prevention or therapy) of allergy illnesses in the future [18]. The authors formulated a sustainable diet based on evidence from observational and interventional studies. This diet should consist of a wide variety of fresh, whole, natural, or minimally processed foods. More than 50% of the diet should be plant-based, including fresh fruits, raw and cooked vegetables, whole grains, legumes, fermented foods, herbs and spices, as well as black and green tea. Additionally, a moderate amount of nuts, peanuts, seeds, omega-3-rich oils, and animal-based products should be included [18]. Recommended foods include those that are high in dietary fiber, fermented foods, and foods that naturally contain beneficial microbes. Additionally, foods rich in flavonoids and polyphenols derived from tea, herbs, and spices are highly regarded as effective anti-inflammatory components, as determined by the Dietary Inflammatory Index (DII) [19,20]. Due to their safety, wide presence in plants, a frequent occurrence in the daily diet, and diverse range of bioactivity, including anti-inflammatory and immunomodulatory properties, polyphenols are considered a valuable and promising dietary intervention for preventing and treating allergic diseases [20,21,22,23]. Consequently, there has been a surge of scientific interest in polyphenols in recent years, leading to significant research. This has been driven by the increasing need for the development of new preventative and therapeutic options using natural ingredients [24,25,26].

This review intends to concisely outline the existing knowledge and advancements in the area of using dietary polyphenols as natural-bioactive compounds for the prevention and treatment of allergic illnesses. We offer an understanding of the mechanisms that contribute to the potential antiallergic properties of phenolic compounds in both experimental and clinical settings. Additionally, we explore the positive clinical effects of polyphenols on food allergies and allergic respiratory diseases. Furthermore, we provide substantial guidance for future research in this area.

### Source of the Data

A thorough literature search for English-language reports was performed from January 2014 to December 2023. Current and relevant data were extracted from Internet sources such as Science Direct, PubMed, Springer, and Google Scholar. The following key phrases were used alone or in combination: allergy, asthma, allergic rhinitis, atopic dermatitis, dietary fiber, dietary lipids, dietary protein, “polyphenols”, “flavonoids”, “flavanones”, “flavonols”, “isoflavonoids”, “anthocyanidins”, “chalcones”, “stilbenes”, “lignans”, and “phenolic acids”. The titles and abstracts were scanned to exclude any unrelated studies. A total of 284 studies concentrating solely on the antiallergic effects of polyphenols on health were searched, and articles having relevant data were examined. Review articles and original research based on animal studies and clinical trials were given top priority.

## 2. Polyphenols and Their Characteristics

**Figure 1:**
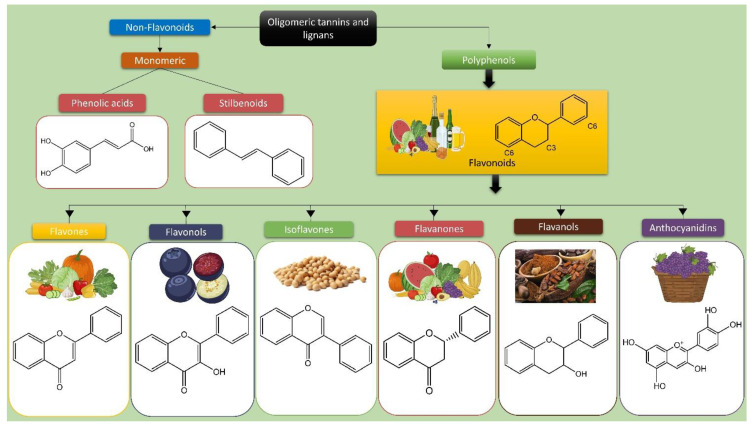
Various polyphenol classes and the chemical structures of their primary constituents.

Polyphenols are a diverse category of more than ten thousand chemical compounds that are naturally found in plants. They are produced as secondary metabolic products in response to free radicals or environmental stress factors [20,27]. Phenolic chemicals serve multiple functions in plants, including defense against pathogens, protection against oxidative damage, acting as light sensors, influencing sensory qualities, and regulating growth and reproduction [28]. Polyphenols are widely found in nearly all plant-derived foods. However, the primary sources of polyphenols in the human diet include fruits, vegetables, seeds, cereals, and nuts. Processed foods including olives, tea, coffee, chocolate, red fermented vinegar, and red wine also contribute to polyphenol intake [29,30]. Polyphenols are categorized into four main types, namely “flavonoids, phenolic acids, lignans, and stilbenes”, based on their structural arrangements that impact absorption, metabolism, bioavailability, and biological activity (Figure 1) [20,28].

Flavonoids, which are the predominant type of polyphenols, may be found in more than 4000 plant species. They are primarily responsible for the vibrant colors observed in leaves, flowers, fruits, and vegetables (Table 1) [31]. The predominant flavonoids that have been extensively researched are quercetin, kaempferol, and myricetin. These compounds are found in significant quantities in kale, onion, tomato, apples, berries, herbal tea, and red wine [32]. Additional significant flavonoids in the diet include isoflavones, which can be found in soybeans; anthocyanidins, which are present in colored vegetables and fruits such as red cabbage, eggplant, berries, and cherries; catechins, which are found in high concentrations in green tea, red wine, and dark chocolate [20]; flavones such as apigenin, luteolin, and baicalin, which are abundant in green and black tea, cereals, and aromatic herbs like celery and parsley [20]; and naringenin and hesperidin, which are phenolic acids, such as gallic, caffeic acid, and ferulic acid, make up almost 30% of the overall polyphenols in our diet [20]. These acids can be found in red fruits, onions, and black radishes [27]. Lignans are a limited group of phenolic chemicals mostly found in linseed, whole grains, and cereals [30]. Resveratrol, a crucial stilbene for human well-being, is mostly found in grape skins, red wine, peanuts, blueberries, and cranberries [33]. However, it is only in the past two to three decades that extensive research has revealed the positive effects of these phenolic compounds on human health. This research has confirmed their ability to combat bacteria and fungi, reduce inflammation, act as antioxidants, modulate the immune system, and provide benefits against diabetes, cancer, blood clotting, and neurodegenerative diseases [20,21,22].

## 3. The Influence of Diet and Nutritional Status on Allergic Reactions

Multiple epidemiological studies have provided evidence indicating that a higher intake of fruits and vegetables is linked to a reduced occurrence of food allergy, allergic rhinitis, and asthma [39,40,41,42,43]. An extensive observational study conducted in children revealed that increased fruit consumption was linked to a decreased incidence of allergic rhinitis, atopic dermatitis, and recurrent wheezing [44]. The study found that children who ingested fruit at least three times per week as part of a traditional diet had a protective impact against wheezing and allergic rhinitis. On the other hand, the consumption of fast food or burgers considerably increased the occurrence of these respiratory conditions [20,44]. Notably, the risk of acquiring allergic disorders was significantly reduced by consuming a diet that includes fruits including apples, pears, carrots, tomatoes, and citrus fruits [45,46]. Independent epidemiological case–control studies conducted in Australia, Finland, and the United Kingdom have demonstrated a strong association between the consumption of apples and pears and a reduced risk of asthma. These investigations also found that individuals who consumed these fruits experienced a lower frequency and intensity of asthma symptoms, as well as a decrease in bronchial hypersensitivity. The findings from these studies were statistically significant [47,48]. In addition, researchers discovered that consuming apples while pregnant acts as a safeguard against the onset of childhood asthma and allergy disorders [49]. A recently published systematic review offers a comprehensive summary of research that examined the effects of dietary therapies on asthma patients. The analysis highlights the most reliable and promising outcomes associated with specific components derived from herbs, herbal mixes, and extracts [50]. The positive effects of preventing allergies that come from consuming a diet that is abundant in fruits, vegetables, and herbs are believed to be due to the high levels of polyphenols, specifically flavonoids, found in these foods [20,50,51].

The Mediterranean diet, known for its high intake of vegetables, grains, and olive oil, has been extensively studied as a dietary pattern that could potentially have a positive effect on the development of asthma and other allergy illnesses [20]. Recent comprehensive evaluations and statistical analyses have yielded highly encouraging findings indicating that following the Mediterranean diet is linked to a lower occurrence of asthma, atopy, and food allergies [52,53]. Furthermore, evidence from both observational and experimental studies indicated that olive oil, which is abundant in polyphenols and fatty acids, plays a significant role in the Mediterranean diet. This dietary pattern has been found to have numerous health benefits, including its notable effectiveness in preventing the onset of asthma and other allergies [54]. A study conducted on a large population verified the link between consuming olive oil and a lower risk of asthma. The study found that for every additional 10 g of olive oil consumed per day, the risk of asthma decreased by an additional 20% [55]. In another study, exposure to resveratrol in the diet during pregnancy was linked to a reduced likelihood of experiencing wheeze and allergic rhinitis [56].

## 4. Polyphenol Bioavailability

In animals, polyphenols are considered xenobiotics and undergo metabolic transformations before being eliminated by animals. However, certain polyphenols can participate in cellular metabolic processes in mammalian tissues, leading to unique therapeutic advantages [57]. Polyphenols undergo metabolism upon oral consumption, and certain polyphenols, including flavonoids, are partially hydrolyzed and occasionally absorbed in the stomachs of monogastric mammals [58]. Typically, the process of hydrolysis, which involves breaking down compounds like polyphenols by removing sugar molecules, takes place in the small and large intestines. This breakdown is facilitated by enzymes found in the intestinal lining and by microbes. It helps the compounds become more easily absorbed and reduces their potential for causing harm [58]. The majority of polyphenols usually reach the colon and undergo conjugation processes (such as glucuronidation) once they are absorbed by the cells lining the intestines. The absorption of the aglycone form of polyphenols in the stomach or small and large intestines, which accounts for approximately 5–10% of the overall polyphenol intake, depends on parameters such as their hydrophobicity or lipophilicity [59,60]. Unabsorbed polyphenols, including those that remain unchanged, are eliminated in feces.

Almost all polyphenols that are taken in by the intestines are carried through the portal vein and undergo glucuronidation, methylation, or sulfation in the liver as a part of the body’s natural detoxification process for foreign substances and these substances undergo conjugation to form polyphenol metabolites. These metabolites can either be transported back to the gastrointestinal tract through the bile duct for further metabolism, or they can be excreted as feces [57]. Alternatively, a smaller amount of aglycone polyphenol metabolites can enter circulation. After being circulated, polyphenols are either eliminated through urine by the kidneys or absorbed into other tissues. Notably, polyphenols that are discovered later in tissues exist in a deconjugated state. This suggests that there is a process of deconjugation occurring inside a living organism, most likely by a process that takes place within the cells, possibly within the endoplasmic reticulum and it may also be initiated by inflammation [61]. When eaten in high concentrations, polyphenols can enter the bloodstream without undergoing significant metabolism and are mostly eliminated in their original form [62]. A study aimed to evaluate the impact of pomegranate polyphenols on the body by administering them through intravenous injection. The results showed that, although polyphenols did accumulate in organs like the heart and brain, they predominantly accumulated quickly in the kidneys after undergoing metabolism in the liver [63].

Differences in gut microbiota and the structure of polyphenols can lead to variations in how polyphenols are metabolized and absorbed in the gut [57,64,65]. Many phytoestrogenic flavonoids, such as genistein, apigenin, kaempferol, and naringenin, are metabolized and absorbed more easily in their aglycone form. However, the hydrolysis and absorption of their glycosidic forms, which are more commonly found in plants, can be more variable and dependent on the types of microflora present [57,66,67,68]. In addition, the absorption of a limited amount of polyphenols in their aglycone form is more easily achieved for specific types of polyphenols, such as procyanidins and catechins [69,70]. Research conducted in a laboratory setting indicates that certain polyphenols in the colon may stimulate the production of conjugation enzymes, such as uridine 5′-diphospho--glucuronlytransferase (UGT) and cytochrome P450 [71]. On the other hand, research conducted in living organisms suggests that the composition of microbiota in the caecum can also affect the ability to produce the enzymes required for conjugation [71,72].

The effectiveness of polyphenol absorption can also be influenced by external factors, such as the composition of the food matrix in which polyphenols are consumed [57], as well as the health status of the individual [73]. The distribution of intestinal microbiota and the metabolism of particular polyphenols, as well as the production of specific conjugated forms, might be influenced by age-related changes and metabolic diseases in the host [74,75]. Polymorphisms in host genes can also impact the metabolism of polyphenols. For instance, the expression of UGT and sulfotransferase enzymes, which are necessary for the conjugation of polyphenolic substances in Phase II metabolism, might be affected by variations in gene polymorphisms among individuals [76,77]. High quantities of polyphenols, whether taken orally or through gavage, or obtained from food containing significant amounts of specific compounds (such as epigallocatechin-gallate in green tea), have been demonstrated to saturate conjugation reactions. This saturation effect results in the increased absorption and circulation of agyclone forms, rather than their conjugated counterparts [78,79,80].

## 5. The Mechanisms behind Allergic Responses

In response to different allergens, all allergy disorders entail type 2 inflammatory reactions. A sensitization and memory phase, followed by an effector phase, characterize the classic allergic reaction [81,82]. Dust mites, fungi, pets, and pollens are common allergens found in the environment [83]. Dendritic cells capture allergens that enter the body through the epithelial barrier, which can be damaged by viruses or other environmental factors, and convey them to naive CD4^+^ T cells during the sensitization phase. This process culminates in the development of allergen-specific CD4^+^ Th2 cells that secrete IL-4, IL-5, IL-9, and IL-13 [82]. The production of three cytokines—TSLP, IL-33, and IL-25—by epithelial cells in response to a threat creates a cytokine milieu that encourages the development of Th2 cells [84]. Stromal cells, similar to epithelial cells, are able to detect changes in metabolite levels and release IL-33 when aberrant metabolite profiles are detected [85,86]. When exposed to high concentrations of IL-4 and IL-13, B cells undergo isotype class switching and develop into antigen-specific plasma cells that secrete relatively larger amounts of IgE. 

Certain innate effector cells, such as mast cells and basophils, have high-affinity FcεRI receptors on their surface, to which IgE binds. Here, B and Th2 cells that are specific to antigens are created as a memory pool [82,83]. Acute effector phase events include allergen exposure, which causes sensitized effector cell IgE cross-linking, effector cell activation, and mediator release (PGD2), including preformed histamine and tryptase, as well as leukotrienes C4 (LTC4), LTD4, and LTE4 [83]. These mediators cause short-lived symptoms in mucosal tissues, including itching, sneezing, coughing, and diarrhea, by interacting with cells of sensory nerves, glands, and epithelium [82,83]. Type I hypersensitivity reactions cause tissue damage and chronic inflammation in the late effector phase due to the accumulation of cytokines IL-4, IL-5, IL-9, and IL-13 produced by Th2 cells and type 2 innate lymphoid cells (ILC2s), as well as cytokines derived from epithelial cells. This keeps antigen-specific IgE levels high and brings in more inflammatory cells, such as eosinophils and basophils, to the inflamed tissue.

While TSLP, IL-33, and IL-25 produced by epithelial cells do more than just serve as alarm signals, they are essential in triggering type 2 immunity [84]. According to the study, they control a variety of immunological cells, such as dendritic cells, which deliver antigens to immature T cells, neuron cells, ILCs, and memory Th2 cells. They also promote the production of Th2 cells, activate ILCs, and stimulate neuronal cells. Thus, it is possible that reducing sensitivity and exacerbations in all allergy diseases can be achieved by targeting these alarmins. Alarmin production can be influenced by one’s diet and diets rich in inulin fiber which, for instance, increase stromal cell IL-33 production via bile acids produced by the gut microbiota [85], whereas diets high in fat increase serum TSLP [87]. Natural flavonoid quercetin reduces TSLP levels in an in vitro atopic dermatitis model using human keratinocytes [88], and dietary fish oil or fermented fish oil (both are rich in long-chain unsaturated fatty acids EPA and DHA) reduces TSLP expression in atopic dermatitis-affected mouse ear tissue [89].

Integrative lymphoid cells (ILCs) are innate immune cells found in tissues; they interact with other cells in tissues, such as neurons, stromal cells, and epithelial cells, to control immunity that is particular to tissues [90]. Type 2 inflammation relies on ILC2 cells, which are abundant in mucosal areas like the lungs, skin, and digestive tract. They play a key role in the development of allergic disorders such as asthma, allergic rhinitis, and atopic dermatitis because they are quickly activated by TSLP, IL-33, and IL25, and they release large quantities of traditional Th2 cytokines IL-4, IL-5, IL-9, and IL-13 [91,92]. By activating the aryl hydrocarbon receptor (AhR), several dietary metabolites can inhibit ILC2 responses, such as retinoic acid, found in carrots, and indole-3-carbinol, found in cabbage and broccoli [91,92]. As a result of these AhR ligands, eating these vegetables may help reduce the risk of allergy-like disorders. In addition to their role as AhR ligands, dietary nutrients can influence ILC2 cells via other pathways. As an example, butyrate, a metabolite of dietary fiber, can limit the proliferation of ILC2 cells and their production of IL-13 and IL-5 by inhibiting histone deacetylase (HDAC). Hence, butyrate, whether administered systemically via water or intranasal injection, can reduce airway hypersensitivity and inflammation caused by ILC2 [93].

Tolerance to allergens and the restoration of immunological homeostasis are achieved through allergen-specific immunotherapy, which relies on allergen-specific regulatory B cells (Bregs) and regulatory T cells (Tregs) [81,82]. A positive CD4^+^ FOXP3^+^ CD25^+^ stain through cell contact-dependent mechanisms or secreted inhibitory cytokines (IL-10, TGF-β), Tregs can suppress persistent allergic inflammation by inhibiting various immune cells and tissues. This includes effector Th cells, granulocytes (including mast cells, basophils, and eosinophils), B cells, and DCs [82]. By exuding suppressive cytokines such as IL-10, TGF-β, and IL-35, which promote Treg formation, limit T cell activation, and stimulate tolerogenic DCs, as well as by producing anti-inflammatory IgG4 antibodies, Bregs also play an important role in preserving tolerance to allergens [82]. Both the production and function of Tregs and Bregs can be impacted by nutrient metabolism. 

Dendritic cells in lymph nodes that drain the nose have a high expression of indoleamine 2,3-dioxygenase (IDO), an enzyme critical for the catabolism of dietary tryptophan to kynurenines and an essential component of immunological tolerance to inhaled allergens. Blocking IDO during intranasal allergen challenge hinders Treg differentiation, which in turn eliminates allergen-specific immunological tolerance [94]. According to research in humans, a reduced IDO level is linked to atopy [95]. Additionally, the metabolic pathways that mothers use to metabolize tryptophan can impact the onset of allergy disorders in their children [96]. Patients with allergic illnesses, such as asthma, allergic rhinitis, or atopic dermatitis, often have a reduced number of regulatory B cells or see alterations in their activity [97,98]. There is a correlation between altered glutamine metabolism and reduced IL-10-secreting Bregs in allergic rhinitis patients [99]. Foxp3^+^ Treg development and the immunological suppression of T helper cells are promoted by both retinoic acid [100] and 1,25-dihydroxyvitamin D3 [101], which are metabolites of vitamin D3. High levels of Th2 cytokines and IgE responses to allergens are induced by dietary vitamin A or vitamin D deficiencies [82,102]. One possible mechanism by which fermented fish oil reduces allergic skin inflammation is by increasing the expression of TGF-γ and IL-10, which in turn may result in the formation of tissue-specific Foxp3^+^ Tregs [89]. Immune tolerance to allergens relies on the trace element zinc, which also encourages Treg differentiation [103,104]. Several antigen-presenting cells express AhR at high levels [105,106], and studies have demonstrated that AhR activation promotes Treg production [107] or IL-10-producing Breg differentiation and function [108] via inducing tolerogenic DC. Dietary supplements containing β-lactoglobulin produced from whey protein complexed with either quercetin–iron or catechine–iron were found to effectively alleviate allergy symptoms in mouse experiments [109,110]. The positive effects have been linked to quercetin- or catechine-activating AhR and -enhanced Tregs [82,109,110].

## 6. Possible Modes of Action for Treating or Preventing Allergy-Related Conditions Using Polyphenols

**Figure 2:**
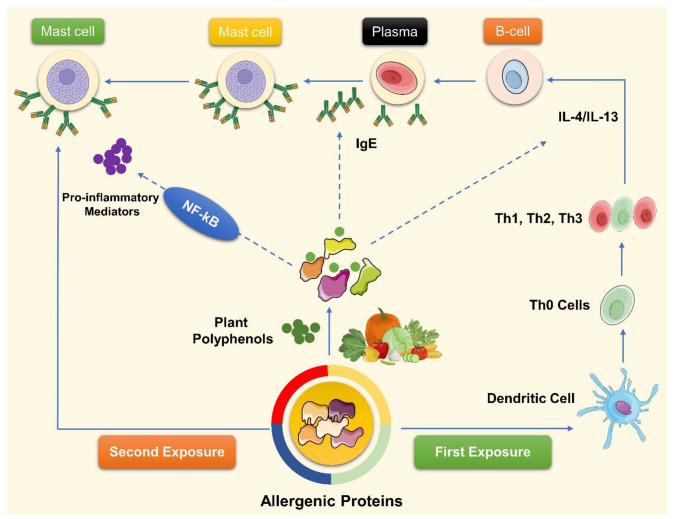
Combining allergenic proteins with polyphenols found in diet can be used to decrease allergy symptoms.

Polyphenols may have preventive and therapeutic benefits for allergic disorders, but the precise molecular and cellular mechanisms by which they do it remain unclear and require further investigation. One possible explanation for polyphenols’ antiallergic effects is that they modulate the immune response both locally and systemically, interacting with allergic proteins to make them less allergenic and affect the diversity and composition of the gut microbiota (Figure 2).

### 6.1. Alteration of Allergenic Protein

Dietary polyphenols can alter the functional characteristics of food allergens through their capacity to generate soluble and insoluble protein–phenolic complexes with altered binding affinities to food proteins [111,112]. Polyphenols and food allergens, whether covalent or non-covalent, may alter the spatial structure of the allergenic protein, which in turn decreases the allergen’s IgE-binding capacity and, by extension, its sensitization potential [112,113]. To reduce allergenicity, certain polyphenols can bind to nucleophilic amino acids to mask linear allergen epitopes and modify the secondary and tertiary protein structure to change the conformational epitopes in allergens [114,115,116]. The main allergen in cow milk, β-lactoglobulin, has been the subject of numerous studies that have shown that, when it is covalently conjugated with different polyphenols, such as rutin, ferulic acid, caffeic acid, epigallocatechin (EGCG), and chlorogenic acid, it causes conformational changes in the protein, leading to a more unfolded structure and a decreased ability to bind IgG and IgE [117,118,119,120,121]. In addition, it has been verified that several flavonoids, including EGCG, naringenin, myricetin, kaempferol, and quercetin, can reduce the allergenicity of β-lactoglobulin through noncovalent interactions. Among these flavonoids, EGCG exhibited the strongest inhibitory effect on β-Lactglobulin antigenicity, leading to a ~75% decrease in the ability of IgE to bind [122] the protein. The allergenicity of ovalbumin, measured in vitro as the ability to trigger the degranulation of effector cells and in vivo as the degree of the allergic immune response and symptom score, is diminished due to the covalent conjugation of quercetin with ovalbumin, that alters the protein’s secondary and tertiary conformation, resulting in a less folded structure and reduced allergen stability [123]. Allergenicity was significantly reduced due to the α-helical structures being lost in the conjugates by as much as 40% and the antigenic epitopes being masked, as revealed by the spectrometric structural analysis of profilin family allergens following both covalent and non-covalent binding with quercetin [124]. Covalent interaction with chlorgenic acid, EGCG, and polyphenols extracted from the Sargassum fusiforme (Hijiki) algae can alter the structure of shrimp tropomyosin, leading to a decrease in allergenicity. This, in turn, alleviates shrimp-induced allergic symptoms in vivo [125,126].

In addition to their structural modification capabilities, polyphenols have the potential to enhance the allergenic potency of proteins in general by modifying their functional properties. This includes digestibility, which is enhanced when more protein cleavage sites are exposed, leading to faster and more effective allergen degradation [127,128]. As an example, research has shown that increasing the digestibility of peanut allergen and modifying its linear and structural epitopes both lead to a dramatic reduction in allergenicity when EGCG and chlorgenic acid are covalently conjugated to peanut proteins [129]. 

In vitro and in the food allergy mouse model, it was found that peanut proteins were less allergenic, as indicated by fewer food allergy reactions, such as symptoms, mast cell frequency, and intestinal damage [129,130,131]. The results for five major apple polyphenols (epicatechin, phlorizin, rutin, chlorogenic acid, and catechin) were similar, proving that polyphenols affect both the spatial structure of Ara h1 peanut protein and the simulated gastric digestion [132]. Interestingly, epicatechin was found to have the strongest inhibitory effect on peanut allergy. In addition to improving the thermal stability and in vitro digestibility of allergic proteins, the covalent binding of wheat gliadin with chlorogenic acid and luteolin affected the IgE/IgG binding capacity by changing the protein conformation and transforming it into a more ordered structure [133,134].

Last but not least, polyphenol–allergen binding may cause protein aggregation and cross-linking, which in turn reduces the allergen load [123,124]. This could be because some reactive allergens are lost and reactive epitopes are less accessible. Conversely, allergen binding is effective when there are fewer polyphenol molecules than allergen reaction sites because polyphenols can form cross-linked protein polymers. This results in more stable and effective polyphenol–allergen complexes [115,135]. Several excellent studies that examined the structural and functional characteristics of soybean globulins following covalent interaction with polyphenols such as EGCG, chlorogenic acid, caffeic acid, gallic acid, and tannic acid [136,137,138] provided clear evidence of this occurrence. There was a decrease in IgE binding activity and histamine release in vitro [136,137,138] as a consequence of structural changes caused by the formation of polyphenol–soybean globulin conjugates and the cross-linking of soybean proteins. These changes also increased UV absorption and protein digestibility. Curiously, studies conducted on an allergic mouse model showed that covalently conjugating the soy 11S protein with EGCG and chlorogenic acid not only reduced the protein’s allergenicity and allergy symptoms but also successfully induced oral tolerance to the soy allergen [138].

Taken together, these findings suggest that dietary polyphenols may help lower food allergenicity, which could lead to the creation of hypoallergenic foods that minimize the symptoms of food allergies or perhaps prevent them from forming in the first place by promoting tolerance.

### 6.2. Actions on the Immune System

How polyphenols can modulate the immune system in cases of allergies has received a lot of research interest of late. Polyphenols can affect the allergy immune response, according to multiple in vitro and in vivo studies [139,140,141,142,143,144,145,146]. They show stimulatory and inhibitory effects at two crucial stages, namely the sensitization and effector phases. Table 2 summarizes the impact of dietary polyphenols on different allergies.

In the initial stage of sensitization, dendritic cells expose the entering allergen to naïve CD4^+^ T cells in draining lymph nodes. This causes the naïve CD4^+^ T cells to differentiate into allergen-specific Th2 cells that produce proallergic cytokines (IL-4, IL-5, IL-9, IL-13) [147]. Research has shown that some polyphenol groups can hinder antigen presentation by influencing dendritic cell maturation, differentiation, and the ability to stimulate T cell differentiation into Th2 cells that are allergic in nature [142]. Resveratrol suppresses dendritic cell maturation, which induces an immature phenotype, and it affects human dendritic cell differentiation from monocytes [148,149]. The capacity to inhibit the phenotypic and functional maturation of dendritic cells produced from murine bone marrow has been established for many polyphenols, including curcumin, fisetin, silibinin, isoflavones, and polyphenols found in blackberries. Additionally, these chemicals reduce the surface expression of MHC class II and co-stimulatory molecules (CD83, CD80, CD86) on dendritic cells, which makes efficient antigen presentation more difficult [150,151,152,153,154]. Additionally, apigenin, EGCG, and other polyphenols were discovered to induce cell death in immature dendritic cells and dendritic cell precursors, as well as to alter dendritic cell differentiation and antigen absorption activity [151,155]. In addition, the priming of naive CD4^+^ T cells, the subsequent crucial step in the sensitization process, might be influenced by polyphenols. The activation and differentiation of naive CD4^+^ T cells into Th2 effector cells can be inhibited by kaempferol and lycoricidine, according to research [156,157].

When epithelial cells lining barrier sites release cytokines like TSLP, IL-25, and IL-33 in reaction to food- and aero-allergens, they activate dendritic cells and innate lymphoid cells type 2 (ILC2), which in turn promote the development of Th2 cells, and they play a significant role in the allergic sensitization phase [158,159]. During the early stages of antigen sensitization, ILC2 plays a crucial role in promoting the Th2 immune response through the production of IL-4, IL-13, and IL-5 [160]. Many polyphenols, including curcumin, baicalin, and quercetin, have been found to inhibit the production of TSLP and IL-33 in atopic dermatitis mice models and human keratinocyte models of the disease [161,162,163]. In a study, authors found that resveratrol and naringenin, two additional polyphenols, suppress TSLP synthesis and messenger RNA expression in human mast cell lines [164,165]. In animal models of allergic airway inflammation, quercetin was found to modulate the cytokines originating from the epithelium. Specifically, it reduced IL-25, IL-33, and TSLP levels in blood samples and the expression of this cytokine in lung tissue [166]. It was reported that the traditional medicinal herb *Fallopia japonica*, also known as Asian knotweed, contains polyphenols like flavones and resveratrol. This herb targets the IL-33/TSLP signaling pathway and significantly lowers the levels of these cytokines in the nasal and bronchoalveolar lavage fluids of mice modeling allergic rhinitis and asthma [167].

During the early stages of sensitization, the proallergic cytokines IL-4 and IL-13 are produced by Th2 and ILC2. These cytokines cause B cells to undergo IgE isotype class-switching and transform into plasma cells. Subsequently, these cells secrete a large amount of allergen-specific IgE, which links to high-affinity FcεRI receptors on the surface of mast cells and basophils, leading to an allergic sensitization state [168]. 

There has not been a comprehensive investigation or description of the influence that polyphenols may have on B cell recruitment, maturation, or function [20,169]. Several polyphenols have been extensively studied in both laboratory and animal settings for their ability to inhibit the production of antigen-specific IgE. These polyphenols include curcumin, rosmarinic acid, quercetin, ferulic acid, tea catechins (including ellagitannins, gallic acid, and EGCG), and polyphenols found in red grapes [170,171,172,173,174,175]. In another study, it was demonstrated how polyphenols can modulate immune responses by utilizing the natural flavonoid dihydromyricetin. This flavonoid successfully inhibited the sensitization phase by lowering B cell numbers, antigen-specific IgE production, and the interaction between FcεRI and IgE [176]. It was also shown that phlorotannins (such as eckol and dieckol) and tea catechins have the potential to bind directly to the α chain of FcεRI. This could prevent antigen-specific IgE from binding to FcεRI and, consequently, reduce mast cell sensitization [177,178,179]. In addition, there is evidence that certain compounds like quercetin, kaempferol, resveratrol, phlorotannins, and saponins can help reduce allergic reactions by lowering the expression of the FcεRI receptor. This receptor is essential for the long-term sensitization of mast cells and their degranulation during the effector phase [179,180,181,182,183].

**Table 2:** Dietary polyphenols have beneficial effects on a range of allergy disorders. A summary of in vitro and in vivo studies.

Polyphenols	Condition	Dose	Study Type	Results/Observations	Reference
Quercetin	Allergic rhinitis	Oral administration of pure isolated polyphenol at doses of 1, 10, and 50 mg/kg	BALB/c mice (in vivo)	Less frequent episodes of stuffy nose, rubbing the nose, and sneezing; reduced production of NO, IgE, and Th2-cytokines	[184]
Quercetin	Allergic rhinitis	20, 35, or 50 mg/kg/day of pure isolated polyphenol	BALB/c mice (in vivo)	Mucus production decreased; lowered serum IgE and histamine levels; decreased inflammatory cell and goblet cell counts in tissues; restricted Th1/Th2 and Treg/Th17 imbalance	[185]
Quercetin	Allergic rhinitis	80 mg/kg of pure isolated polyphenol	Sprague–Dawley rats (in vivo)	Lesser secretion, sneezing, and itching; reduced synthesis of IgE and Th2-cytokines; reduced number of eosinophils in the nasal turbinates mucosa	[186]
Luteolin	Allergic rhinitis	10, 30 mg/kg of pure isolated polyphenol	PBMC (in vitro) BALB/c mice (in vivo)	Reduced allergic reactions and HDM-specific IgE levels in blood; IL-4 production inhibition	[187]
Naringenin	Allergic rhinitis	100 mg/kg of pure isolated polyphenol	Sprague–Dawley rats (in vivo)	Reduced levels of serum total IgE, IL4, and IL5; decreased shedding of skin cells, erosion, and the presence of eosinophils in the nasal lining	[188]
Resveratrol	Allergic rhinitis	5, 30, 50 mg/kg of pure isolated polyphenol	BALB/c mice (in vivo)	Reduced levels of histamine, specific-IgE, IL-4, IL-5, IL-13, IL-17, and inflammatory cell counts (leukocytes, eosinophils, lymphocytes, and neutrophils)	[189,190]
Resveratrol	Asthma	30 mg/day of pure isolated polyphenol	BALB/c mice (in vivo)	Suppressed OVA-triggered inflammation in the airways and reduced mucus secretion	[191]
Resveratrol	Asthma	100 mg/kg of pure isolated polyphenol	C57/Bl16 mice (in vivo)	Decreased inflammation and infiltration of eosinophils	[192]
Resveratrol	Asthma	100 mg/kg of pure isolated polyphenol	C57BL/6J mice (in vivo)	Protecting bronchial epithelial cells from oxidative DNA damage and apoptosis when exposed to HDM allergen	[193]
Curcumin	Asthma	10, 20 mg/kg of pure isolated polyphenol	BALB/c mice (in vivo)	Reduced airway inflammation and free radical damage; stimulated Treg cells	[194]
Curcumin	Asthma	120 mg/kg of pure isolated polyphenol	BALB/c mice (in vivo)	Decreased cytokine production of IL-4, IL-5, and IL-13; reduction in tissue eosinophilia and excessive mucus production	[195]
Curcumin	Asthma	800 mg of pure isolated polyphenol	BALB/c mice (in vivo)	Reduction in pulmonary inflammation; Marked decrease in eosinophils and excessive development of goblet cells; reduced production of Th2-related cytokines IL-4, IL-5, and IL-13, as well as Th17 cytokine IL-17A	[196]
Luteolin	Asthma	0.1 mg/kg of pure isolated polyphenol	BALB/c mice (in vivo)	Substantial reduction in IL-4, IL-5, and IL-13 levels in the lung homogenate and in the infiltration of inflammatory cells in lung tissue	[197]
Epigallocatechin gallate	Asthma	20 mg/kg of green tea extract	BALB/c mice (in vivo)	Decreased asthmatic symptoms, lung inflammatory cell infiltration, inflammatory factors, and increased Treg proportion	[198]
Gallic acid, ellagic acid	Asthma	100, 300 mg/kg	BALB/c mice (in vivo)	Decreased levels of inflammatory cytokines, IgE, and inflammatory cell count; Decrease in the movement of inflammatory cells and the production of mucus in lung tissue	[199]
Epigallocatechin, epigallocatechin gallate	Food allergy	50 mg/day extracted from tea	BALB/c mice (in vivo) model of αs1-casein milk protein allergy	Histamine, specific IgE antibodies, mast cell protease, and Th2 cytokines were all markedly decreased; mild alterations in intestinal pathology	[200]
Curcumin	Food allergy	3 mg, 30 mg/kg of * Curcuma longa * extract	BALB/c mice (in vivo) food allergy model	Reduced incidence of food-induced reactions, including hypothermia and anaphylaxis; enhanced cytokine production by Th1, decreased production by Th2, and inhibited IgE; ensuring a steady ratio of Th1/Th2	[201]
Resveratrol	Food allergy	2.5–40 μg/mL 5, 10, 20 mg of * Abies georgei * extract	RBL-2H3 cells (in vitro) and BALB/c mice (in vivo)	Degranulation of mast cells and the release of histamine and β-hexosaminidase are decreased; reducing the likelihood of diarrhea increases the regulation of rectal temperature; lower concentrations of histamine, mouse mast cell protease-1, and specific IgE in serum	[202]
Baicalin	Food allergy	50, 100, 200 μmol/L 20 mg/kg of * Scutellaria baicalensis * extract	Caco-2 cells (In vitro) and BALB/c mice (in vivo) food allergy model	Decrease in food allergy symptoms, serum IgE levels, and Th2 cells that promote allergic responses; increased expression of regulatory T cells; improving intestinal barrier function by controlling tight junctions	[203]
Anthocyanidins	Food allergy	1 and 5 mg/mL of wild blueberry extract	Caco-2 cells (in vitro)	Improvement in the intestinal barrier function and maintenance of the integrity of the intestinal cell monolayer; decreased gut permeability, higher transepithelial electrical resistance (TEER), elevated expression of claudin-1	[204]
Ferulic acid caffeic acid apigenin luteolin	Food allergy	1–3 g/kg/day of olive oil	BALB/c mice (in vivo)	Restored the structure of the ileum villi and increased the production of tight junction proteins; elevated levels of Treg-associated cytokines (IL-10) in the lamina propria; reduced levels of Th2 cell-related cytokines in the lamina propria; decreased Burkholderiaceae and increased Clostridiaceae in the gut microbiota	[205]
Catechins	Food allergy	0.05% 0.1% areca nut extract via drinking water	BALB/c mice (in vivo)	Reduced allergic reactions triggered by OVA, such as diarrhea; diminished penetration and release of granules from mast cells in the duodenum; inhibited the development of particular IgE and Th2 immune response	[206]

When the same allergen is re-exposed during the effector phase, it causes the surface of mast cells and basophils to become inflamed and produce reactive mediators, which generate an acute systemic allergic reaction [158]. The processes by which polyphenols may modulate mast cells, the primary effector cells of an allergic response, have recently been the subject of a plethora of in vitro and in vivo investigations [144]. Polyphenols like resveratrol, quercetin, and procyanidins from cinnamon or apple extract can inhibit the cross-linking of IgE by allergens on the surface of mast cells, thus suppressing their activation [207,208], in addition to the effects on the expression of the FcεRI receptor and the FcεRI-IgE binding that have already been mentioned. Numerous polyphenols, such as quercetin, phlorotannins, luteolin, and myricetin, have been shown to stabilize mast cell membranes and inhibit degranulation by downregulating the expression of calcium channel proteins and blocking the influx of calcium and the elevation of intracellular calcium levels required for mast cell degranulation [209,210,211]. Indeed, curcumin, resveratrol, rosmarinic acid, and these phenolic compounds all showed strong inhibitory effects on histamine and β-hexosaminidase release. These enzymes are used to measure the extent of mast cell degranulation [212,213,214]. Furthermore, polyphenols have been found to effectively inhibit the signaling cascades of both FcεRI-mediated protein kinases (Syk, Lyn, PLCγ, PKC) and the MAPK and the NF-κB signaling pathways, which play a crucial role in allergic reactions. This, in turn, reduces the production of pro-inflammatory cytokines (IL-4 and TNF-α) and lipid mediators (prostaglandin D2 and leukotrienes) [215,216,217]. Honey from bamboo and rubber trees, which are rich in polyphenols, showed an inhibitory effect on mast cell activation and degranulation, confirming the anti-allergic potential of stingless bee honey according to research [218]. Honey from noni and mango trees, which are poor in polyphenols, did not exhibit any anti-allergic action.

During the later effector phase, there is an overexpression of Th2-related immune response and an increase in the production of Th2 cytokines, such as IL-4, IL-5, and IL-13. This leads to many consequences, including the maintenance of high levels of antigen-specific IgE, the recruitment of immune cells like eosinophils to inflammatory sites, an increase in mucus production, and the initiation of chronic allergic inflammation, which damages and remodels tissues [219]. 

Many studies in both animal and cellular models have shown that polyphenols inhibit Th2 differentiation, downregulate Th2-related cytokine production, reduce inflammatory cell infiltration, and suppress allergic inflammation via their immunomodulatory effects at multiple critical stages of the effector phase. By increasing Th1 pathways and decreasing the upregulation of Th2-mediated immune responses, polyphenols successfully restore the Th1/Th2 imbalance [23,139,169]. The anti-allergic action of curcumin has been demonstrated in various models of allergic diseases. This action is believed to be due to the following mechanisms: a decrease in IL-4, IL-5, and IL-13 secretion; an inhibition of the activation and infiltration of macrophages, monocytes, neutrophils, and eosinophils into inflammatory sites; and a shift in the Th1/Th2 response towards the Th1 phenotype [220,221]. By reducing the synthesis of IL-4, Il-5, and Il-13 in serum and bronchoalveolar lavage fluid (BALF), kaempferol and rosmarinic acid alleviated airway inflammation, which in turn reduced eosinophil recruitment into lung tissues, airway hyperresponsiveness, and hyperproduction of mucus [222,223]. This effect was especially observed in asthma models. According to research conducted in a mouse model of allergic rhinitis, flavonoids like quercetin, isoquercetin, myricetin, and luteolin reduce inflammation in the nasal mucosa. This is achieved by promoting the Th1 pathway and maintaining a balance between Th1 and Th2, as well as by suppressing cytokine secretion and Th2 cell differentiation [224,225,226,227,228]. Furthermore, in mouse models of food allergy, quercetin and tea catechins (gallic acid and ellagitannins) inhibited the ovalbumin (OVA)-induced allergic response, promoting immune tolerance through Th1/Th2 modulation and Treg induction [229].

The disruption of the equilibrium between Th17/Treg cells, together with Th1/Th2 dysregulation, aids in the destruction of immunological tolerance and, by extension, plays a role in the development and worsening of chronic allergic inflammation [230]. New experimental evidence suggests that flavonoids such as baicalin, cyanidin, quercetin, and luteolin can reduce allergy reactions by balancing the Th17/Treg ratio and increasing the number of regulatory T cells (Tregs) [231,232]. Similarly, curcumin reduced Th17 cell differentiation and increased Treg subtype numbers in a mouse asthma model, suggesting a modulatory impact on the Th17/Treg imbalance [233,234,235].

### 6.3. Application of Polyphenols in Dietary Allergies

The effects of different polyphenols on the immune response, which controls the consequences of an allergic reaction, have been the subject of a great deal of research in animal models of food allergies, with some suggesting that these compounds may alleviate food hypersensitivity and allergy symptoms in sensitive mice [24]. According to the study, in a model of mice sensitized by αs1-casein milk protein, tea catechins like epigallocatechin (EGC) and epigallocatechin gallate (EGCG) effectively suppress mast cell activation, specific IgE and Th2 cytokine production, and pathological alterations in the intestine to a lesser extent [200]. Polyphenols found in Chinese sweet tea, specifically ellagitannins and gallic acid, have been shown to effectively suppress allergic responses in mice when exposed to hen egg ovalbumin [20]. This suppression was achieved through alterations in the Th1/Th2 balance, an increase in the percentage of the Treg subtype, and an improvement in intestinal IgA secretions. As a result, the mice showed fewer symptoms like itching, lethargy, and gastrointestinal signs [172]. Resveratrol, myricetin, quercetin, curcumin, and other polyphenols derived from apples and areca nuts have also been shown to have a significant therapeutic impact on food allergies. These polyphenols not only reduce rectal temperature and alleviate diarrhea and anaphylactic reactions, but they also suppress the allergic response by preventing mast cell infiltration and degranulation in the duodenum, lowering serum levels of specific IgE, reestablishing a Th1/Th2 imbalance, and increasing the population of Treg [202,206,236]. It is worth noting that a rat study on food allergies found that, when cocoa-derived flavonoids were given to the animals at the same time as an allergen during the induction phase, they were able to inhibit both the local and systemic immune response [237]. This was demonstrated by the suppression of Th2-related cytokines released from spleen cells and a mesenteric lymph node, which led to a protective effect against food allergies. However, this protective effect was not enough to prevent anaphylactic reactions after an oral allergen challenge [237].

Furthermore, multiple studies have shown that polyphenols can influence the local immune response. This includes reducing intestinal Th2-mediated immunity, altering TCR-mediated signaling cascades, and promoting Treg cell differentiation and function in lamina propria. The result is the preservation of immune tolerance, which is closely linked to the development of oral tolerance and the reduction in food allergies [238,239,240,241]. Specifically, research has shown that eating foods that are high in polyphenols, like cocoa flavonoids or apple-condensed tannins, can reduce the body’s sensitivity to an oral allergen and ward off food allergies. The reason behind this protective effect is an upregulation of the percentage of TCR T cells, the primary subset of intraepithelial T lymphocytes, which is essential for building immune tolerance [239].

The main risk factors for food allergies are known to be an impaired intestinal barrier and increased intestinal permeability. Intolerance begins when a dietary antigen breaches the gut epithelial barrier and is transformed into peptides by antigen presentation cells (APCs). These peptides are then presented on the surface of APCs for recognition by antigen-specific Th cells. Since dietary polyphenols can improve the integrity and function of the intestinal barrier, they may be able to prevent or reduce the symptoms of food allergies by inhibiting the permeation of allergens. Indeed, research in both laboratory and animal settings has shown that consuming polyphenols in the diet can improve the function of the intestinal barrier and decrease the amount of water that can pass through. This is achieved through various pathways, such as increasing the expression of the intestinal tight junction protein, increasing the transepithelial electrical resistance (TEER) across a cellular monolayer, decreasing oxidative stress, and blocking signaling pathways involving NF-κβ and MAPK, which are involved in inflammation [242,243,244]. 

Quercetin, luteolin, naringenin, kaempferol, curcumin, grape seed proanthocyanidin, wild blubbery anthocyanins, chlorogenic acids, and green and black tea flavonoids have all been studied at concentrations ranging from physiological (e.g., epicatechin) to pharmacological (e.g., berberine) to determine their potential beneficial effects on intestinal barrier function and integrity and, by extension, the relief of food allergy symptoms [245,246,247,248,249]. One of the key components of the Mediterranean diet, olive oil, contains a high concentration of polyphenols such as phenolic acids (ferulic and caffeic), lignans, and flavones (apigenin, luteolin) [54]. Recent studies have shown that polyphenols can regulate intestinal immunity and improve intestinal barrier function, which in turn prevents food allergies [20]. Research on animals has shown that taking an olive oil supplement can alleviate food allergy symptoms like itching and gastrointestinal distress. It also strengthens the intestinal barrier by mending damaged ileum tissue villi, increasing the expression of TJ protein, and lowering mucin production. Furthermore, olive oil upregulated the Treg population and increased intestinal sIgA production, which in turn promoted the development of antigen tolerance and the maintenance of intestinal immunity [205,241]. It also reduced the Th2-cytokine level in lamina propria and the degree of tissue inflammation. It has been shown that adding polyphenols from apples and red wine to the starter diet of weaning piglets can affect the structure and function of the gut barrier. They have also found that these polyphenols can suppress the activation of GALT in Peyer’s patches in the ileum, which means that the piglets experience less intestinal inflammation and develop immune tolerance more quickly [169,250].

**Figure 3:**
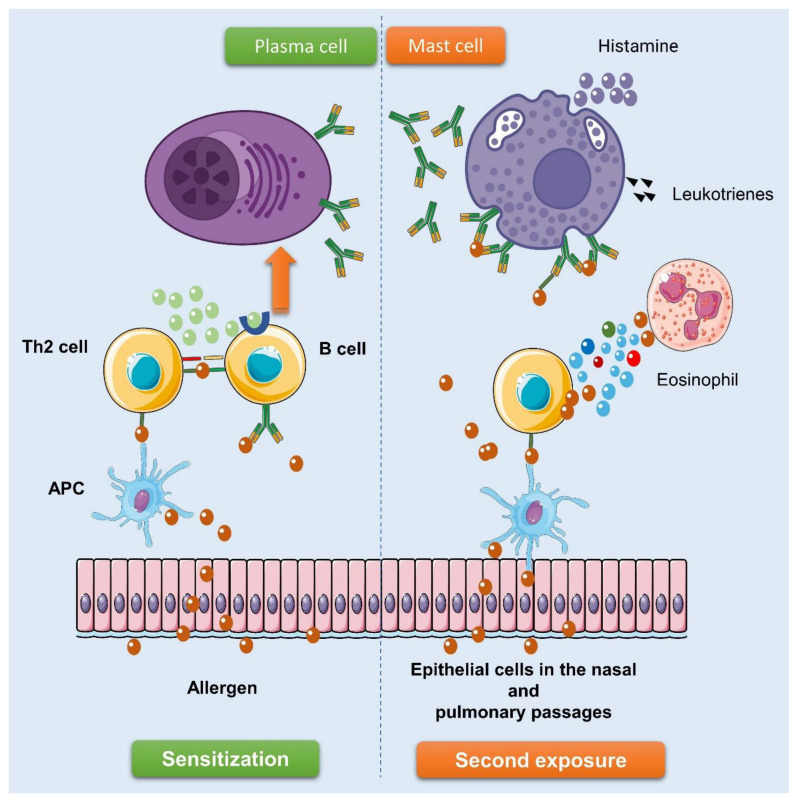
A diagrammatic illustration of the systems that polyphenols interact with to influence the immune response in food allergies.

In conclusion, there is a wealth of evidence from animal studies and in vitro experiments to suggests that polyphenols can modulate the allergic sensitization process and potentially prevent the development of allergic diseases. Additionally, their effect on allergy effector cells during re-exposure suggests they could be a new therapeutic strategy. The polyphenol mechanisms that modulate the response to food allergy could be summed up as follows: (i) potential interactions between polyphenol allergens and the digestive tract and bioaccessibility; (ii) interactions between polyphenol allergens and the epithelial barrier may affect absorption and transport across the barrier; (iii) Polyphenols can improve the functioning of epithelial cells and can also impact the abnormal regulation of T cells; and (iv) APC and IgE antigen recognition could be hindered by polyphenol allergen interactions (Figure 3).

More clinical trials are required to confirm their effects in human patients, but investigating dietary phytochemicals and their metabolites for anti-allergic potential is an exciting new avenue for basic research. Recent clinical intervention trials in allergic illnesses indicate the therapeutic effects of dietary phytochemicals [251,252], as shown in Table 3. Patients with allergic rhinitis experienced a marked improvement in their symptoms after 14 days of taking a new barley-based formulation. This formulation outperformed fexofenadine in managing nasal congestion, postnasal drip, and headache [253]. Possible explanations for this helpful impact on allergy symptom control include the soluble fiber and phytochemicals included in barley drinks.

## 7. Future Challenges and Restrictions

There has been a plethora of encouraging evidence from recent investigations about the benefits of polyphenols on allergic disorders. Prior to incorporating the existing information into dietary or therapeutic recommendations, there are a number of obstacles and limits to overcome in the study of polyphenols’ biological effects, especially in humans.

Since the polyphenol content of food and vegetables varies greatly, it is hard to achieve the required biologically active concentrations in vivo by food alone [20,57]. Secondly, it is very difficult to compare the effects of various polyphenols found in food sources and to provide accurate recommendations regarding the most beneficial foodstuff. This is due to the high degree of variability and the lack of information regarding the exact concentration of polyphenols in food and data on the polyphenol concentrations achieved by their actual consumption. 

Hence, additional studies are required to determine and describe polyphenolic compounds’ natural sources, to standardize polyphenolic extracts to a point that they can be used as therapeutic agents, and, most crucially, to identify and delineate the antiallergic effects of active phenolic components and metabolites.

Additional in-depth research analyzing the mechanism of action, degree of activity, and structure–activity relationship are necessary. This is because polyphenols exert a wide spectrum of biological effects. This is also indicative of the fact that a combination of polyphenols may lead to a more effective beneficial effect [267]. 

Further investigation into polyphenols is necessary regarding their administration methods, target tissues, optimum doses, and the optimal composition of phenolic extracts. To establish the maximum safe single dose and long-term safety profile, preclinical studies must test a wide range of doses. Polyphenols, due to their natural origin, are generally believed to be non-toxic and safe. Data from clinical and preclinical studies demonstrate that the evaluated phenolic compounds have good tolerability, no adverse effects, and high safety [20,267,268]. This, however, cannot be established as a rule. To evaluate the total toxicity and content of harmful compounds produced during food processing or polyphenol extraction, more research is required.

Regarding polyphenol’s capacity to lessen allergenicity by interacting with allergic proteins, it is worth noting that how polyphenols and proteins in food bind in function of the processing methods used, and can involve both covalent and non-covalent interactions [20,111]. Covalent allergen-polyphenol conjugates, which are generated in an alkaline environment or through enzymatic oxidation, are often preferred over non-covalent conjugates, which are formed in acidic or neutral environments, due to their irreversibility and stability [269]. Furthermore, environmental variables including temperature, ionic strength, and salt concentration might impact the polyphenol–protein interaction [20,114].

Since the amounts of phenolic compounds in the blood after consumption are greatly affected by several circumstances, the bioavailability of polyphenols poses a significant barrier to the investigations into their efficacy in both allergic animal models and humans with allergic conditions [18,20]. Polyphenol bioavailability is limited by weak chemical stability, quick and widespread metabolism in the liver and intestinal epithelium, and the kind of dietary source, all of which affect polyphenol absorption in the intestines [270]. The breakdown of polyphenols into their microbial derivatives by the gut microbiota further complicates their absorption, bioavailability, and bioactivity, which may vary from the parent molecule [271]. There is a lack of data on the function of these metabolites at this time; additional research is required to assess their possible function and to clarify the two-way connection between microbiota and polyphenols.

In addition to studying ways to increase the bioavailability of phenolic compounds, future studies should look for a food source that guarantees the adequate absorption of natural plant polyphenols. To improve the bioavailability and effectiveness of polyphenols, various drug delivery systems have recently been studied, with encouraging outcomes [272,273,274]. These systems include lipid-based carriers, polymer nanoparticles, and conjugate-based systems.

Interactions with other bioactive chemicals in the food matrix can alter the bioaccessibility, bioavailability, metabolism, and biological effects of polyphenols [275]. When analyzing the outcomes of studies that primarily examined the positive impact of a single phenolic component, it is crucial to include these interactions along with the presence of other bioactive molecules in the diet. Because there is known inter-individual variability in reactions to phenolic chemicals based on the dietary pattern [20,22], this caution also applies to data from clinical investigations. In addition, different people may have different reactions to polyphenols since their microbiota and metabolic conditions are unique, which is influenced by the differences in metabolic enzyme activity [276]. Future studies should thus take these inter-individual differences, along with the possible impact of age and ethnicity, into consideration.

There is an immediate need for large-scale, carefully planned clinical trials in humans and population studies to assess the therapeutic utility of various polyphenolic substances in respiratory allergies. According to the aforementioned findings, there is a significant increase in the prevalence of allergic disorders. The prevalence of allergies or allergic disorders is influenced by various factors that govern the population’s vulnerability to acquiring allergic symptoms. Allergy incidence in individuals is mostly determined by a combination of genetic and environmental factors. Approximately 10% of the world’s population is affected by various allergy illnesses, including mild rhinitis, severe anaphylaxis, or asthma [277]. Table 4 provides a concise overview of the frequency and reasons behind various allergies.

## 8. Conclusions and Take-Away Message

Allergies are a significant issue that impacts millions of individuals worldwide. Avoiding exposure to an allergen that is uncommon or unknown can be challenging and may lead to unintentional exposure. Allergic individuals can, nonetheless, reduce symptoms by abstaining from contact with allergens. Presently, the existing diagnostic and therapeutic approaches focus on mitigating symptoms; nevertheless, medicine does not offer enduring alleviation from allergic conditions. Researchers are currently performing novel research and inquiries in order to discover effective remedies for the treatment of allergies. As our understanding of the connection between nutrition and allergic diseases continues to expand, more and more research is focusing on the potential anti-allergic effects of natural food components. These components have the potential to improve both the dietary and therapeutic approaches to managing allergic disorders. Dietary polyphenols have recently gained attention as the most comprehensive class of bioactive secondary metabolites. These compounds have a wide range of biological effects, including antioxidant, immunomodulatory, and anti-inflammatory capabilities. However, when polyphenols are ingested in large quantities without medical supervision, whether as dietary supplements or plant extracts, the danger of undesirable side effects increases. When persons who are being treated for various chronic conditions (such as renal damage, cancer, thyroid disorders) and must take prescription medications on a daily basis consume a polyphenol-rich diet or supplements, the negative effects of polyphenols are also increased. Therefore, it is critical to raise public knowledge about the potential negative effects of polyphenol supplementation, particularly in the case of various susceptible populations. After reviewing the available research, it is clear that polyphenols have a lot of promise as a preventative measure (functional foods or supplements) or a therapeutic intervention (in the context of allergic illnesses). For polyphenols to be widely used as pharmaceutical agents or dietary interventions, more research is needed to understand their bioavailability, metabolic differences between individuals, and possible mechanisms of action. Nevertheless, the data that are available now offer promising future prospects. Since the public is becoming more health-conscious and more likely to self-medicate with supplements, it is reasonable to assume that polyphenol-rich foods will continue to be a regular part of people’s diets and be used to create functional foods and supplements.

## Figures and Tables

**Table 1:** Primary categories of polyphenols with antiallergic characteristics and their corresponding dietary sources.

Polyphenol Class	Food Source	Reference
Flavonols	Grapes, onions, kale, tomatoes, broccoli, fennel, pickles, okra, rocket, tea, red wine, beer, cocoa, bee pollen, and berries (cherries, apricots, cranberries, and grapes)	[23,32]
Flavones	Orange skin and pulp, lemon peel and pulp, green pepper, artichoke, cabbage, watermelon, melon, cantaloupe, apples, green tea, and black tea	[34,35]
Isoflavones	Soybeans, black beans, and green peas	[23,36]
Flavanones	Seeds, citrus, and tomato peels; mint and chamomile	[30,37]
Flavanols	Grape peels, apple, grape, and seed peels, toasted peanuts, almonds, pistachios, leaves of green tea rosemary, red wine, and chocolate	[23,30]
Anthocyanidins	Berries, bananas, strawberries, cherries, pears, figs, plums, beans, red cabbage, and grape skins and wine lees	[30,34]
Phenolic acids	Onions, black radishes, red fruits, citrus peels, grapes with seeds, potato skin peel, tea, and coffee	[20,27]
Stilbenes	Red and white wine, berry fruits, strawberries, and grape byproducts	[20,33]
Lignans	Strawberry, peach, oat, wheat, rye, barley, cabbage, broccoli, garlic, olives, and grains and cereals	[30,38]

**Table 3:** The role of various polyphenols in diverse allergic conditions.

Health Condition	Major Polyphenol	Study Design	Outcome/Result	Reference
Allergic rhinitis	Rosmarinic acid at doses of either 50 mg/day or 200 mg/day	21-day randomized, double-blind, placebo-controlled study with 29 participants	A notable rise in responder rates for all symptoms associated with seasonal allergic rhinoconjunctivitis	[254]
Allergic rhinitis	Quercetin with kaempferol glycosides at a dosage of 100 mg per day	12-week randomized, double-blind, placebo-controlled trial, conducted with 39 participants	A notable difference in the symptom score and in the symptom plus medication score for Japanese cedar pollinosis 10 weeks post-intervention	[255]
Allergic rhinitis	Naringenin at a dosage of 360 mg per day	8-week randomized, double-blind, placebo-controlled study with 33 participants	A marked reduction in the overall index of symptoms associated with chronic allergic rhinitis	[256]
Allergic rhinitis	Quercetin at a dosage of 100 mg per day	8-week randomized, double-blind, placebo-controlled research including 20 participants with a therapeutic design	Japanese cedar pollinosis is associated with a marked improvement in ocular symptoms	[257]
Allergic rhinitis	Silymarin at a dosage of 420 mg per day	1-month trial with 60 participants, conducted with randomization, double-blinding, and placebo control	A substantial reduction in the intensity of allergic rhinitis symptoms	[258]
Atopic eczema or dermatitis	Quercetin	There were 15 human participants diagnosed with contact dermatitis. Topical use of quercetin for five days	No difference compared to the control group	[259]
Atopic eczema or dermatitis	Cocoa flavanols, including catechin, epicatechin, and procyanidins, in doses of either 27 mg or 329 mg	Ten healthy women ingested a low and high dosage	A higher dosage of cocoa drink enhanced blood circulation to the skin and decreased water loss	[260]
Atopic eczema or dermatitis	A quercetin-rich water extract from whey powder dodder	A randomized controlled trial (RCT) study enrolled 52 patients with atopic dermatitis for 30 days	Quercetin decreases mast cell production and lowers allergic symptoms; improve the skin’s suppleness and hydration levels	[261]
Atopic eczema or dermatitis	Apple-condensed tannins (ACT) administered at a dosage of 10 mg/kg	8-week study examined the effects of apple polyphenols on individuals with atopic eczema	Diminished inflammation and itching in patients with the illness in comparison to the control group. ACT exhibits an anti-allergic effect	[262]
Asthma	500 mg/day of ellagic acid	64 participants with mild to moderate allergic asthma	Enhanced clinical manifestations of asthma such as day dyspnea, nocturnal dyspnea, and reduced asthma-related activities; decrease in eosinophil, basophil, and neutrophil levels	[263]
Asthma	Curcumin at a dosage of 30 mg/kg/day	Individuals with chronic asthma aged 7–18 years	Enhanced disease management: reduced nighttime disturbances and decreased reliance on short-acting β-adrenergic agonists	[264]
Asthma	Curcumin at a dosage of 1000 mg daily	77 patients with mild-to-moderate bronchial asthma	Substantial enhancement in the average FEV1 values	[265]
Asthma	A blend containing 100 mg of pycnogenol and water-soluble bioflavonoids.	76 patients with asthma	A decrease of 15.2% was observed in the specific IgE levels, but the levels of IgG1 and IgG4 were constant; decreased medication requirement	[266]

**Table 4:** Allergy symptoms, causes, and prevalence among the global population.

Type of Allergy	Symptoms	Prevalence	Affected Organ	Causes	Reference
Allergic rhinatisis	Symptoms such as a stuffy or runny nose, red, itchy, watery eyes, and swelling around the eyes may be present	Impacts anywhere from 10% to 30% of the global population	Nose	Genetic and environmental factors	[278]
Asthma	A tightness in the chest, wheezing, coughing, and difficulty breathing	Impacts anywhere from 3% to 9% of the global population	Airways of lungs	Genetic and environmental factors	[279]
Food allergy	Swelling of the tongue, itching, vomiting, low blood pressure, difficulty breathing, rashes, and diarrhea	Impacts 8% of the world’s population	Skin, respiratory system, abdomen, and retina	Immune response to food	[280]
Skin allergy	Redness, swelling, rash, flaking or scaling skin, broken skin, elevated bumps	Lifetime prevalence rates above 20% on a global scale	Skin	Poison oak, poison ivy, latex, food, medications, water, sunlight, nickel, chemicals, and soap	[281]
Drug allergy	Inflammation of the face, itching, rash, fever, hives, difficulty breathing, and cardiac symptoms	Has an impact on 10% of the global population	The nasal passages, bronchi, larynx, ear, stomach lining, and top layer of skin	Side effects of medications	[282]
Insect allergy	Redness, swelling, itching, and discomfort at the bite or sting site or in the areas immediately surrounding it	There has been no comprehensive documentation of the many severe cases of allergies associated with insect bites that have been documented worldwide	Faces, eyes, neck, and tongue	Insects bite or sting	[283]
Anaphylaxis	Irritation, tingling, numbness, swelling of the throat, lightheadedness, and difficulty breathing	Has an impact between 0.05 and 2% of the global population	Nose, skin, throat, lungs, and gasinterstitial space	Medications, foods, and insect bites	[284]

## Data Availability

Data sharing is not applicable.
